# Video-based feedback as a method for training rural healthcare workers to manage medical emergencies: a pilot study

**DOI:** 10.1186/s12909-017-0975-3

**Published:** 2017-08-31

**Authors:** Zainab Oseni, Hla Hla Than, Edyta Kolakowska, Lauren Chalmers, Borimas Hanboonkunupakarn, Rose McGready

**Affiliations:** 1 Shoklo Malaria Research Unit, Mahidol-Oxford Tropical Medicine Research Unit, Faculty of Tropical Medicine, Mahidol University, Mae Sot, 63110 Thailand; 2 Mahidol-Oxford Tropical Medicine Research Unit, Faculty of Tropical Medicine, Mahidol University, Bangkok, 10400 Thailand; 3 Centre for Tropical Medicine and Global Health, Nuffield Department of Medicine, University of Oxford, Oxford, OX3 7FZ UK

**Keywords:** Feedback, Video, Direct observation, Self-assessment, Confidence

## Abstract

**Background:**

Video-based feedback has been shown to aid knowledge retention, skills learning and improve team functionality. We explored the use of video-based feedback and low fidelity simulation for training rural healthcare workers along the Thailand-Myanmar border and Papua New Guinea (PNG) to manage medical emergencies effectively.

**Methods:**

Twenty-four study participants were recruited from three Shoklo Malaria Research Unit clinics along the Thailand-Myanmar border and eight participants from Kudjip Nazarene Hospital, PNG. The teams were recorded on video managing a simulated medical emergency scenario and the video was used to aid feedback and assess performance using Observed Structured Clinical Examination (OSCE) scoring and Team Emergency Assessment Measure (TEAM) questionnaire. The process was repeated post-feedback at both sites and at 6 weeks at the Thailand-Myanmar border site. Thailand-Myanmar border participants’ individual confidence levels and baseline knowledge (using OSCE scoring) were assessed before team assessment and feedback at week 1 and repeated post-feedback and at 6 weeks. Focus group discussions (FGD) were held at each Thailand-Myanmar border clinic at week 1 (8 participants at each clinic).

**Results:**

Individual paired tests of OSCE scores showed significant improvement post-feedback at week 1 (*p* < 0.001) and week 6 (*p* < 0.001) compared to baseline OSCE scores. There was a trend for increased team OSCE scores compared to baseline at week 1 (*p* = 0.068) and week 6 (*p* = 0.109) although not significant. Thailand-Myanmar border TEAM scores demonstrated improvement post-feedback mainly in leadership, teamwork and task management which was sustained up to week 6. PNG showed an improvement mainly in teamwork and task management. The global rating of the teams’ non-technical performance at both sites improved post feedback and at week 6 on the Thailand-Myanmar border site. Self-rated confidence scores by Thailand-Myanmar border participants increased significantly from baseline following training at week 1 (*p* = 0.020), and while higher at 6 weeks follow up than at baseline, this was not significant (*p* = 0.471). The FGD revealed majority of participants felt that watching the video recording of their performance and the video-based feedback contributed most to their learning.

**Conclusion:**

Video-assisted feedback resulted in an improvement in clinical knowledge, confidence and quality of teamwork for managing medical emergencies in two low resource medical facilities in South East Asia and the South Pacific.

**Electronic supplementary material:**

The online version of this article (doi:10.1186/s12909-017-0975-3) contains supplementary material, which is available to authorized users.

## Background

South East Asia has one of the highest deficits of healthcare professionals with a density of 4.3 per 1000 patients, significantly lower compared to 18.9 per 1000 in Europe [1]. Thailand has a ratio of 0.3 doctors per 1000 patients and although 65.7% of the Thai population live in rural areas, only 16.5% of doctors work in rural hospitals, compared with 28% of nurses and 20.4% of rural primary care workers [2]. In Myanmar, the doctor to patient ratio is 0.3:1000 whilst the nurse/midwife to patient ratio is 0.8:1000 [3]. The deficit is much greater in Papua New Guinea (PNG) where the doctor to population ratio is 0.06 per 1000 whilst the community health worker to population ratio is about 0.6 per 1000 and the nurse to population ratio is 0.5 per 1000 [4]. Hence, management of acute illnesses invariably falls on the shoulders of primary care workers and nurses who act as middle level care providers to plug the gap between healthcare need and healthcare service provision.

Although various studies have identified lack of training as a reason for poor emergency medical care provision in low resource settings [5–7], there is a dearth of literature about methods of training rural healthcare workers to be competent in dealing with emergency medical illnesses. This is worrying given that the biggest causes of mortality in these regions are malaria, diarrhoea and pneumonia, and these can all present as medical emergencies. Furthermore, as non-communicable diseases become more prominent in low to middle income countries, acute illnesses such as heart failure, diabetic and hypertensive emergencies will become increasingly common [8]. It is, therefore, essential to provide effective training for rural healthcare workers in low resource settings to enable them to manage acute illnesses appropriately.

Simulation-based training has been used extensively in the field of medicine. It enables participants to learn from their mistakes and accommodates focused, repetitive practice which allows participants to develop the desired skills and behaviors [9, 10]. Simulation exposes gaps in knowledge in areas such as teamwork and practical procedures which are difficult to assess through written or oral assessment. It also improves confidence [10]. Debriefing is an essential part of simulation-based training which enables students to reflect on their experience and incorporate it into meaningful learning [10–12].

Video-assisted feedback has long been used in different areas of medicine as well as outside the field of medicine in areas such as disaster management and in the air force [13–24]. It is a method where participants’ performance of a task is recorded and feedback is given with the aid of the recording. Video-assisted feedback provides objective evidence of an individual’s performance because it provides accurate, real time data that cannot be disputed thereby enabling feedback [25, 26]. It also highlights inconsistencies between participants’ perception of their performance and their actual performance which, according to learning theorists, improves skills [25]. Indeed, studies have shown improvement in clinical skills following video-assisted feedback [27, 28]. In addition, video-assisted feedback speeds up learning [28]. Scherer et al. [28] found that participants who received video feedback for trauma resuscitation performed better after 1 month compared to those who got verbal feedback for 3 months. Furthermore, video-assisted feedback aids knowledge retention, reduces anxiety and improves team functionality [13, 25, 29, 30]. Even without instructor feedback, video-assisted feedback has been shown to be an effective training tool when used for self-assessment by participants [27, 30, 31]. However, some studies have reported no difference in effectiveness of video-assisted feedback compared to oral feedback [31, 32].

The current pilot study builds on work from a previous study which looked at the use of a structured ABCDE approach to train rural staff in three clinics along the Thai-Myanmar border [33]. Whilst the previous study relied on didactic teaching and the Observed Structured Clinical Examination (OSCE) method of assessment with verbal feedback, we explore the use of video-assisted feedback and low fidelity simulation as a means of training rural healthcare workers in Thailand and PNG to manage medical emergencies effectively.

The aim of the current study is to determine if the use of video-assisted feedback is an effective means of training rural healthcare workers in Thailand and Papua New Guinea (PNG) to manage medical emergencies effectively.

## Methods

### Settings ﻿(Fig. 1)

#### Thailand-Myanmar border

The three Shoklo Malaria Research Unit clinics; Mawker Thai, Mae La and Wang Pha, are located along the Thailand-Myanmar Border and were selected for inclusion in the training. Shoklo Malaria Research Unit provides mainly maternal and neonatal care in Mae La, the largest refugee camp in Thailand, with an estimated population of 45,000. The main health provider in Mae La camp at the time of this training was Première Urgence – Aide Médicale Internationale [33]. Wang Pha and Mawker Thai clinics provide more general basic health care for the Burmese and Karen migrant population, and have adult inpatient and outpatient care facilities in rural and remote locations. Over a 6 month period from February 2015 to July 2015, Mae La had 12 medical emergencies, Mawker Thai had 7 and Wang Pha had 16.

**Fig. 1:**
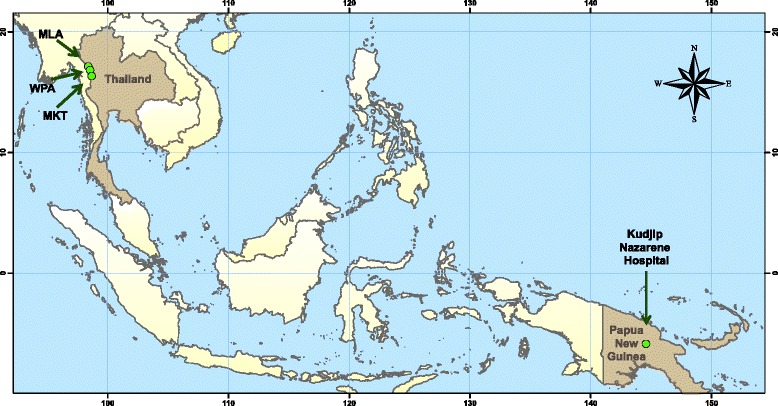
Map of the study settings (created using ArcGIS 10.2 by Daniel Parker)

In the last decade, the prevalence of *P. falciparum* malaria in the migrant and refugee population has declined steeply and with this, the number of cases of severe malaria requiring resuscitation [34]. Therefore, staff exposure to managing the acutely comatose, or convulsing medical emergencies has reduced significantly. Sepsis and obstetric and neonatal emergencies commonly present to the clinics [33].

#### PNG highlands (PNG)

Kudjip Nazarene Hospital is located in the highlands of PNG. It provides paediatric, medical, surgical and obstetric services. Although the hospital provides outpatients services for patients from all provinces of PNG, it only provides inpatient care for patients residing in Jiwaka province which has a population of 343,987 [35]. In 2015, 57,599 patients were seen in the outpatient clinic and there were 6291 admissions. A large proportion of patients present with medical emergencies, commonly sepsis.

### Study participants

#### Thailand-Myanmar border

The study was explained to Shoklo Malaria Research Unit staff at clinic sites 1 week ahead of starting the study to allow them to consider taking part in the study. The idea of training for emergencies with video was explained to them as a group in their native language, emphasizing that participation was voluntary.

#### PNG

A ‘medical emergencies’ training session was organised and the Emergency Department staff were invited to participate voluntarily.

At both sites, there was no risk involved for participants and no incentive or compensation was provided to participants. Confidentiality has been maintained by de-identifying responses and changing participants’ names.

### Developing the tool

A previous pilot study was carried out by Stanley et al. 2015 [33] to teach rural clinic healthcare workers at Shoklo Malaria Research Unit’s three clinics to manage emergency scenarios using the ABCDE approach. Based on the limitations of the pilot study, especially the cultural barriers to feedback in Asia (*‘loss of face’*) [36], the use of video simulation as a means of improving quality of care was broached.

Rather than just say what action they would take; participants would mime their actions. Therefore, a mannequin was provided as well as a table with equipment and medication required for an emergency medical situation. For instance, if the participants chose to give IV fluids, they were required to pick up the right type of fluid and put it next to the mannequin and this would be videotaped.

The training material was tailored to equipment and medication available at the sites. For example, in the pulmonary oedema scenario, the Shoklo Malaria Research Unit participants were only required to give frusemide as glyceryl trinitrate spray and morphine are not available at the clinics.

Clinical scenarios were written by the trainer and sent to physicians experienced in working locally, for feedback; most of whom also had experience of working in emergency medicine. Some of the scenarios were modelled directly on cases observed at the Shoklo Malaria Research Unit Mawker Thai clinic.

The training sessions were limited to 8 participants at each site to make training and assessment of participants manageable for trainer and assessors. Furthermore, the small number allows each participant to contribute to the team management of the clinical scenario.

At Shoklo Malaria Research Unit, the level of training of participants was kept the same at each site as follows; 4 medics, 2 nurses, 1 junior and 1 senior midwife. They were selected following staff discussion to avoid disruption of routine work due to less staff members on the clinic floor and to determine those who could commit for the full length of the study. A ‘medic’ usually undergoes six to 12 months of theoretical education and 1 year internship whilst the nurses undergo 3 months theoretical education and three to 6 months of internship. Internships involve learning by working in a clinical setting under a supervisor.

Additionally, the amount of training given at each site was varied to ascertain how many sessions were needed to maintain knowledge at 6 weeks. One training session was delivered at Mae La (week 1), two sessions at Wang Pha (week 1 and 3) and four sessions at Mawker Thai (Weeks 1, 2, 3 and 4).

At Kudjip Hospital, only 1 training session was delivered and there were 3 nurses, 1 healthcare assistant and 4 nursing students in attendance. These participants were attending or were certified from a government certified training school with the exception of the healthcare assistant who has no nursing training.

### Training and assessment

The assessors and trainer have Advanced Life Support training and each worked more than 4 months in emergency medicine in Europe. However, week 6 follow up assessment in Wang Pha was carried out by a doctor trained in Thailand due to one of the assessors becoming ill. For the Shoklo Malaria Research Unit training, a translator fluent in English, Burmese and Karen was present for assessment at week 1 and week 6. At Kudjip Hospital, all participants were fluent in English.

Participants were trained and assessed by OSCE using an ABCDE approach which is a systematic approach to assess an acutely unwell patient starting with the airway (A), followed by breathing (B), circulation (C), disability (D) and exposure (E), consecutively. This approach was agreed by the medical faculty in the previous pilot study. Therefore, the scoring form used by the previous pilot study was adapted slightly to fit the scenarios being used for training in the current study whilst still maintaining its ABCDE structure (Additional file 1).

#### Thailand-Myanmar border

On week 1, assessment of individual participants’ baseline knowledge was carried out using scoring form described above. The pass mark was 14 out of 26 but participants failed the scenario if they did not perform certain actions or perform dangerous actions such as giving IV bolus to a patient with pulmonary oedema. Individual feedback was not given at this point.

Immediately following the baseline assessment, the participants were brought together and videoed managing the same scenario as a team. They were allocated 10 min. Recording was done using a cheap compact camera (£80 GBP). Team performance was assessed using the same scoring form used to assess individual performance. The video was replayed for the team and discussed; focusing on what went well, and what could have been improved. The discussion was facilitated by the trainer who also gave feedback and addressed gaps in the knowledge of the team identified from watching the video and the discussion. The same scenario was then repeated and videoed. The second recording was watched by participants and discussed briefly and feedback was given on second performance. This was also scored using the OSCE form. Following this, individuals were assessed again using the same scoring sheet. Individual feedback on performance was given at this point. All recordings were later reviewed by the trainer and used to score teamwork using the Team Emergency Assessment Measure (TEAM) questionnaire which assesses leadership, teamwork and task management [37] (Additional file 2).^3^

Focus group discussions (FGD) were facilitated using a semi-structured interview to ascertain whether participants benefited from the training in terms of knowledge and confidence, which aspect of the training contributed the most to their development and to get general viewpoints of participants about the day. One session was held at each of the three clinics along the Thai-Myanmar border with 8 participants at each clinic. Themes arising from the FGD were identified and coded using Thematic Analysis.

Participants were asked to rank their confidence before and after the training session using a questionnaire with a 5-point Likert Scale to assess confidence in managing an emergency using the ABCDE approach. With a score of one representing the lowest level of confidence up to five for highest level of confidence. The questionnaire is based on a similar one used in the pilot study by Stanley et al. 2015 [33] described above.

Duration of training and assessment including focus group discussion, at week 1, was about 4 h in total at each site. Training sessions at weeks 2, 3 and 4 took on average 45 min and consisted of videoing the participants managing the scenario, discussion and feedback and then repeating the same process. A different scenario was used for training each week. No handouts were given at the end of each session.

Follow up assessment of individuals and teamwork was carried out at week 6 on the same participants at all 3 sites. This included an assessment of confidence levels. The scenario used for the follow up assessment was different from those used for training.

#### PNG

Participants were videoed managing a medical emergency scenario as a team to assess their baseline knowledge. Again, this was done using a cheap compact camera. Team performance was assessed using the same OSCE scoring form. The video was replayed for the team and discussed, focusing on what went well and what could have been improved. The discussion was facilitated by the trainer who also gave feedback and addressed gaps in the knowledge of the team identified from watching the video and the discussion. The same scenario was then repeated by the team and videoed. The second recording was watched by participants and discussed briefly and feedback was given on second performance. This was also scored. The pass mark was 14 out of 26 but participants’ failure to perform certain key actions or perform dangerous actions resulted in a ‘no pass’ result. Participants were also asked to individually rank their confidence levels before and after the training. The recordings were later reviewed by the trainer and teamwork was scored using the TEAM questionnaire.

### Statistical analysis

SPSS version 23 for Windows was used to compare the results of proportions using the Chi-squared test and the t-test and Mann–Whitney-U test for comparing parametric and non-parametric data, respectively. For staff with paired data from consecutive assessments the Wilcoxon signed rank test was used to compare the OSCE and confidence scores.

## Results

### Participants and sociodemographic at Thailand-Myanmar border and PNG

**Fig. 2:**
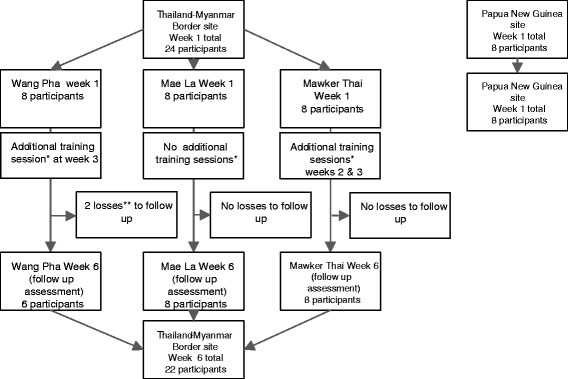
Study flow chart

The available demographic data for the Thailand-Myanmar Border and Papua New Guinea (PNG) were summarized (Table 1) and the participants available at each time point presented in the study flow chart (Fig. 2). The previous pilot study by Stanley et al. 2015 [33] was attended by 6 of the 8 (75%) of participants at Maela, 4 (50%) at Mawker Thai and 5 (62.5%) at Wang Pha.

**Table 1:** Participant demographic

Characteristics	Thailand-Myanmar border *N* = 24	Kudjip (PNG) *N* = 8
Median Age (range) in years	31 (24–45)	28.5 (22–40)^b^
Job description		
Medic	12	-
Midwife	6	-
Nurse	6	3
Student nurse	-	4
Health-care assistant	-	1
Site		
Mae La	8	-
Mawker Thai	8	-
Wang Pha	8	-
Number of years at current job		
< 1 year	0	a
1–5 years	9	a
> 5 years	15	a
Number of years since qualification		
Not applicable	-	5^c^
> 1 year	2	-
1–5 years	12	-
> 5 years	9	2
no reply^d^	1	1
Place of Training		
Shoklo Malaria Research Unit	20	-
Nazarene college of nursing (PNG)	-	7
Other	4	1^e^

^a^information not obtained from participants

^b^only 4 people provided their ages

^c^4 nursing students and 1 healthcare assistant who have no qualifications

^d^1 midwife at Thailand-Myanmar border site and 1 nurse at PNG site gave no reply

^e^healthcare assistant has no formal qualification

### Individual and team OSCE assessment

The median individual OSCE assessment scores for each site were summarized for baseline, week 1 and week 6 assessments (Table 2). Significant improvement at week 1 post feedback and at 6 weeks compared to baseline scores were observed using individual paired tests. There was a trend for increased team scores after baseline although this was not significant (Table 2). PNG team was not assessed at 6 weeks.

**Table 2:** OSCE assessment result, median of individual scores and of teams at each site

Site	Participants at each training (pre, post, 6 weeks)	Scores, median [range], out of 26			*P*-value (paired test)^	
		pre-training week 1	Post training week 1	Follow up week 6	Pre-Post	Pre-week 6
Individual scores average						
Mae La	8, 8, 6	17.5 [16–22]	25 [22–26]	25 [24–26]	0.012	0.027
Mawker Thai	8, 8, 8	13 [5–19]	23 [13–16]	26 [23–26]	0.012	0.012
Wang Pha	8. 8. 8	16.5 [8–23]	24 [18–26]	24 [21–25]	0.011	0.012
Total Thailand-Myanmar border	24, 24, 22	17 [5–23]	24.5 [13–26]	25 [21–26]	<0.001	<0.001
Team scores						
Mae La	1	17	26	26	0.068	0.109
Mawker Thai	1	19	26	26		
Wang Pha	1	19	25	24		
PNG	1	12	18	n.a.		

^*P*-value Wilcoxon signed rank test

n.a. not available

### TEAM scores

Thailand-Myanmar border TEAM Scores as assessed by the trainer demonstrated that improvement 1 week post-baseline training was mostly in the areas of leadership, teamwork and task management and this improvement was sustained up to the follow up assessment at 6 weeks post-baseline (Table 3). Global rating of the team’s non-technical performance also improved post training at week one and at week six.

**Table 3:** Team Emergency Assessment Measure (TEAM) questionnaire

Category		Pre-training			Post-training week 1			Post-training week 6		
		Mawker Thai	Mae La	Wang Pha	Mawker Thai	Mae La	Wang Pha	Mawker Thai	Mae La	Wang Pha
Leader-ship	The team leader let the team know what was expected of them through direction and command	0	1	1	4	4	4	4	4	3
	The team leader maintained a global perspective	0	1	0	4	4	4	4	4	4
Team work	The team communicated effectively	3	2	2	4	4	4	4	4	4
	The team worked together to complete the tasks in a timely manner	4	3	4	4	4	4	4	4	4
	The team acted with composure and control	4	1	3	4	4	4	4	4	3
	The team morale was positive	3	4	4	4	4	4	4	4	4
	The team adapted to changing situations	2	4	4	4	4	4	4	4	4
	The team monitored and reassessed the situation	3	4	3	4	4	4	4	4	4
	The team anticipated potential actions	1	4	3	4	4	4	4	4	4
Task Management	The team prioritised tasks	1	3	2	4	4	4	4	4	4
	The team followed approved standards and guidelines	3	4	2	4	4	4	4	4	4
Overall	On a scale of 1–10 give your global rating of the team’s non-technical performance	4	6	5	10	10	9	10	10	9

0 = Never/ Hardly ever; 1 = Seldom; 2 = About as often as not; 3 = Often; 4 = Always/Nearly always

PNG TEAM scores showed an improvement in the areas of teamwork and task management. The global rating of the team’s non-technical performance also improved after training. The team leadership quality was high both before and after the training (Table 3).

### Confidence

**Fig. 3:**
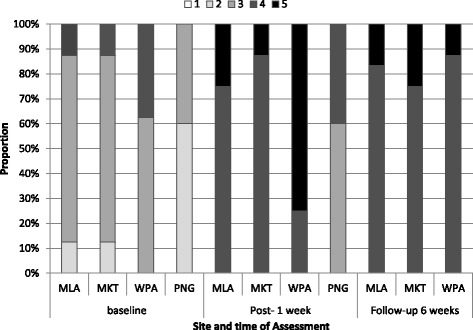
Confidence levels at all study sites. Footnote: 1 = not confident through to 5 = extremely confident

Confidence scores for the baseline and 6 week follow up comparison were only available for Thailand-Myanmar border group. Confidence scores rated by the teams themselves increased significantly from baseline to 1 week post baseline (*p* = 0.020), and while higher at 6 weeks follow up than at baseline, this was not significant (*p* = 0.471) (Fig. 3).

### Focus group discussion

Selected comments from the focus group discussion are presented according to the themes identified including knowledge, confidence, leadership and teamwork (Additional file 3, Table 4). The majority of participants mentioned that learning how to apply a systematic ABCDE approach practically to emergencies was the main thing they learnt from the training session. They highlighted the practical nature of the training as a contributor to their increased knowledge. Some participants pointed out that the session was useful for reminding them what they had been taught previously. Other participants mentioned specific knowledge gaps which were highlighted by the training session and there were positive comments about feedback.

**Table 4:** Selected responses from focus group discussion

Category	Type of Response	Responses
Knowledge	Filled out gaps in theoretical and practical knowledge	Before the training, I could not go step by step” “Before training I didn’t know we need to give the IV steroid directly” “Now I learnt something new. I can give IV fluids. Previously I didn’t know how much”
	Served as a reminder	“I haven’t had training for a long time so I forgot. So now I remember” “I know the ABCD emergency procedure but I couldn’t remember. Like breathing; how can [I] check breathing? How can [I] listen? but after the training, I know this more”
	Reasons for increase in knowledge	“Now we have practical experience so I know more than [I did] previously” “Now we have the chance to do things practically so [it feels] real”
Confidence	Reason for increase in confidence	“After this session, I have more confidence because I know how to go step by step” “I didn’t know the step by step approach but now I know from this training. I have more confidence.”
Which aspect of training contributed the most to learning	Watching video was helpful	“The video is the most important and the most effective because we watched the video clip [of] what we did the first [time] and then saw our weak points and strong points then we repeated it again so we could see which part we improved…. we can see directly” “when we saw the video, we knew how we’re doing and if we’re missing something and the next time we try to correct [ourselves] ………. Like I put the Guedel in the wrong position like that. [laughs]” “I notice that the first time we were standing there not very active but the second time I felt like [we were] more alive like in a real situation. so it’s good to see the comparison; to see ourselves.”
	Watching video and video-assisted feedback was helpful	“After watching the video and receiving the feedback, we understood” “Feedback is good- feedback after watching the video” “The practical approach, the teamwork and also the video and also the feedback”
Leadership	The role of a leader	“If we have a good team leader, we can perform very well and we have confidence to perform well”
	Who should lead an emergency scenario?	“Something I want to ask; who is the leader? We don’t know…” “I think the leader should be more experienced. If people have been here longer, they know more, and they can communicate” (*another participant disagreed*) “The leader should be more experienced in medical emergencies, not just someone who’s worked here for a long time”
Teamwork	Importance of teamwork	“We like the team approach. Previously, we were not taught using a team approach….” “We worked as a team so we cannot forget”

Almost all the participants felt more confident about managing emergency scenarios after the training session. Again, they generally felt that learning how to use a systematic approach was the reason for their boost in confidence. In Mae La, the participants generally felt that they could each lead an emergency scenario. They also highlighted the importance of leadership in emergency medical situations.

Majority of the participants identified watching the video recording of their performance and the video-assisted feedback they received as the parts of the training session which contributed most to their learning. Only one person cited the individual feedback during the post assessment as the most important contributor to his learning. Several of the participants highlighted the teamwork approach of the training as important for their learning.

Finally, although, many of the participants said they would like more training to practice other emergency scenarios, they felt that they would be able to apply what they learnt during the training session in other emergency scenarios.

## Discussion

To the best of our knowledge, there have been no previous studies investigating the use of video-based feedback as an effective means of training in medical emergencies to mid-level providers in low resource settings. The use of video-based feedback in the current study is based on the premise that firstly, previous studies in various areas of medicine including emergency medicine have shown this to be an effective method of training for improving clinical skills [25–28]; and secondly, video devices are cheap and easy to obtain.

At Shoklo Malaria Research Unit, we demonstrated an increase in all participants’ individual OSCE scores from baseline immediately post training at week 1. Furthermore, at Shoklo Malaria Research Unit, most participants maintained or scored higher than the post training score at week 6 follow up irrespective of the number of sessions received after the week 1 training. For instance, Mae La participants had only one training session but 5 of the 6 participants present for the week 6 retest maintained or scored higher than their week 1 post-training scores.

There was also an increase in team OSCE scores immediately post initial training at both Shoklo Malaria Research Unit and Kudjip Hospital. Week 6 retest scores were higher than baseline pre-training scores at all 3 Shoklo Malaria Research Unit sites. However, the increase in team scores both immediately and at 6 weeks follow up in Shoklo Malaria Research Unit was found not to be significant. At week 6, Mae La scored higher than Wang Pha and the same as Mawker Thai despite having only one training session. It, therefore, appears that an increase in number of training sessions given over the 6 week periods did not correlate with increase in scores at retest. It is possible that this effect was a result of Mae La being the stronger group in terms of baseline knowledge and this is certainly reflected in the average baseline OSCE score but interestingly not in baseline team score. Furthermore, the Mae La team have a greater cumulative number of years of work experience compared to the other two teams.

The confidence levels of all participants at both Shoklo Malaria Research Unit and Kudjip Hospital also increased immediately post training at week 1, which is typical of most training [10]. At Shoklo Malaria Research Unit, at week 6, it was still higher than their baseline levels. However, participants at Wang Pha lost some confidence compared to their week 1 post-training levels despite having one additional session. This might reflect the fact that they were tested on a different scenario to their previous training scenarios and so did not feel as confident managing their retest scenario but this does not explain why this did not happen at the other 2 sites. The improvement in participants’ confidence has implications for patient care. In one study, nurses considered self-confidence essential for effective clinical decision making [38]. Another study found that self-efficacy, which is closely related to self-confidence, helps to predict motivation and performance [39]. Therefore, it is helpful to improve self-confidence through training strategies that reinforce positive feedback to improve self-efficacy.

We not only assessed clinical knowledge, we also assessed the quality of teamwork because teamwork is an important determinant of the quality of patient care given by staff [40]. A literature review reported that teamwork has a significant role in the causation and prevention of adverse events in dynamic domains of healthcare such as emergency medicine and resuscitation teams [40]. At Shoklo Malaria Research Unit, there was an improvement in teamwork quality, most marked in the area of leadership immediately post-training at week 1 which was sustained up to the follow up assessment at week 6. Similarly, at Kudjip hospital, in most respects, the quality of teamwork improved following training albeit more modest than the improvements gained in Shoklo Malaria Research Unit. Of note, their skill in monitoring and reassessing situations did not improve following training. One possible reason for this is that, unlike the Shoklo Malaria Research Unit participants, prior to this training, the Kudjip hospital participants had no previous training using the ABCDE approach to manage medical emergencies. The improvement in the quality of teamwork at both sites is likely due to participants having the chance to watch themselves acting out the scenario which potentially helped them to become more self-aware. They were able to observe how they work as a team and how they perform as an individual in the team and this would have made it easier to determine how to improve their teamwork [13, 25, 29, 30]. This is supported by a comment made by one of the participants during the focus group discussion: “I notice that the first time we were standing there not very active but the second time I felt like [we were] more alive like in a real situation. So it’s good to see the comparison; to see ourselves.”

Since the training consisted of various aspects, not just video-based feedback, we recognised that there could be other factors that contributed to improvement in clinical skills such as the quality of training and feedback [41]. The focus group discussion at Shoklo Malaria Research Unit provided insight on what the participants felt was useful. The majority of the participants mentioned the use of video as the biggest contributor to their learning. However, although not stated as the biggest contributors, people mentioned that practicing as a team and the practical approach was useful for building their confidence and for learning.

Although it does not pay attention to the team’s scores, a team approach in a culture that is sensitive to be exposed to personal feedback, can help improve individual performance and confidence. Therefore, it was felt that the video-based approach would aid feedback in a culturally sensitive way in both South-East Asian and Melanesian cultures where *“saving face”* is important and feedback could often be construed as criticism. Indeed, this was found to be the case as participants could see their strengths, weaknesses and errors directly and point them out themselves as opposed to having the trainer point them all out. Moreover, they seemed open to discussing these errors and even sometimes pointing out their own individual mistakes and laughing at themselves. The expression of the teamwork approach of the training as important for learning indirectly prevents individual ‘loss of face’ which is an important aspect of feedback in Asian cultures [42].

There are limitations to the current study. Firstly, we did not include a control group who did not receive video based feedback. This would have given more insight into whether using video-based feedback as opposed to feedback on its own resulted in the improvement in performance. Secondly, some of the improvement in week 1 immediately following training could have been partly due to content specificity as participants were assessed using the same scenario used for training. Research based evidence suggests content specificity is a significant cause of unreliability in assessment research [43, 44]. However, there was sustained improvement in performance at the 6 week follow up in spite of a different scenario being used for follow up assessment. Furthermore, it would have been ideal to have a longer assessment interval to ascertain if skills are retained over a longer period. There were two occasions where the videotaping device failed which means that parts of scenarios were “lost” and so we couldn’t watch these parts during the feedback or during the scoring using the TEAM tool.

## Conclusion

We demonstrated that video-assisted feedback resulted in an improvement in clinical knowledge, confidence and quality of teamwork for managing medical emergencies in two low resource medical facilities in South East Asia and the South Pacific. This improvement was maintained by most participants at 6 weeks follow up irrespective of the number of sessions they received during this period. Furthermore, this method is particularly useful as a sensitive way to give feedback in cultures where saving face is important. Participants stated that they found the use of video-based feedback to be useful for their learning. Therefore, video assisted feedback appears to be an effective method for training staff to manage medical emergencies in low resource settings.

## Additional files

Additional file 1: OSCE scoring form for assessing ABCDE approach to medical emergencies management. (DOCX 14 kb)

Additional file 2: TEAM questionnaire for assessing quality of teamwork in managing medical emergencies. PDF file (.pdf) 269 KB. Detailed information on the tool can be accessed from http://medicalemergencyteam.com/ (PDF 268 kb)

Additional file 3: Focus Group Discussion (DOCX 13 kb)

## Abbreviations

FGD: Focus Group Discussion
OSCE: Observed Structured Clinical Examination
PNG: Papua New Guinea
TEAM: Team Emergency Assessment Measure
