# Supplementary material for: Video-based feedback as a method for training rural healthcare workers to manage medical emergencies: a pilot study
Source: BMC Med Educ. 2017 Aug 31;17:149. doi: 10.1186/s12909-017-0975-3 (PMC5580284; doi:10.1186/s12909-017-0975-3)
Supplement: Supplementary file 1 — OSCE scoring form for assessing ABCDE approach to medical emergencies management. (DOCX 14 kb) [file 12909_2017_975_MOESM1_ESM.docx]

**Participant name___________________Site______________Date__________________ Pre|_| or Post|_|**

**AIRWAY**

| *Candidate assessment/ action* | *Score* | |
| --- | --- | --- |
| Airway clear? Speaking? +/- airway maneuvers ** | 1 |  |
| Airway adjunct (in reduced consciousness scenario) | 1 |  |
| Total |  |  |

**BREATHING**

| *Candidate assessment/ action* | *Score* | |
| --- | --- | --- |
| **Respiratory Rate **** | /1 |  |
| **Auscultate lungs** | /1 |  |
| Chest movements ?symmetry  Tracheal tug?  Accessory muscle use?  cyanosis  Trachea central | /3 max |  |
| Sit patient up (in resp scenarios) | /1 |  |
| **O2 Saturations **** | /1 |  |
| Oxygen high flow (>10 L) ** | /1 |  |
| Total |  |  |

**CIRCULATION**

| *Candidate assessment/ action* | *Score* | |
| --- | --- | --- |
| **Pulse ** (also rhythm and strength)** | /1 |  |
| **Blood pressure /capillary refill (in child) **** | /1 |  |
| **Auscultate heart** | /1 |  |
| Check urine output/ fluid balance/catheter | /1 |  |
| IV line inserted | /1 |  |
| Lie patient flat (in low BP scenarios) | /1 |  |
| **Fluids- correct fluid ****  **-correct amount **** | /1  /1 |  |
| **Scores 2 for NOT giving fluid bolus in pulmonary oedema scenario and fails test if gives bolus)**** | /2 |  |
| **Temperature **** | /1 |  |
| Total |  |  |

**DISABILITY /4**

| *Candidate assessment/ action* | *Score* | |
| --- | --- | --- |
| **Conscious level (AVPU) **** | /1 |  |
| **Blood sugar **** | /1 |  |
| **Dextrose ** (if dextrose normal in scenario, ask what participant would do if dextrose were low)** | /1 |  |
| Pupils (size and reaction to light) | /1 |  |
| Total |  |  |

**EXPOSURE/EVERYTHING ELSE /6**

| *Candidate assessment/ action* | | *Score* | |
| --- | --- | --- | --- |
| Head to toe/front and back exam - Include abdominal examination | | /1 |  |
| **Calls for HELP at any time in scenario **** | | /1 |  |
| **REASSESS after interventions **** | | /1 |  |
| Correct diagnosis | | /1 |  |
| Correct medication given | Asthma- peak flow, steroids, salbutamol neb (needs all 3 to get point) | /1 |  |
|  | Sepsis- ceftriaxone | /1 |  |
|  | Pulmonary oedema- frusemide | /1 |  |
|  | Hypoglycaemia + severe dehydration  (automatically gets 1 point) | /1 |  |
| Total | |  |  |

Please circle:

| Total score | /26 | Pass | Fail |
| --- | --- | --- | --- |

**= Vital step. If missed out ANY then overall fail, (Though still want full score documented - Need >14 and all yellow boxes, to pass)

Abbreviations : AVPU= simple conscious level assessment where A=alert, V=voice, P=pain, U=unresponsive

BP= Blood pressure; PR=pulse rate
