# Supplementary material for: Video-based feedback as a method for training rural healthcare workers to manage medical emergencies: a pilot study
Source: BMC Med Educ. 2017 Aug 31;17:149. doi: 10.1186/s12909-017-0975-3 (PMC5580284; doi:10.1186/s12909-017-0975-3)
Supplement: Supplementary file 2 — TEAM questionnaire for assessing quality of teamwork in managing medical emergencies. PDF file (.pdf) 269 KB. Detailed information on the tool can be accessed from http://medicalemergencyteam.com/ (PDF 268 kb) [file 12909_2017_975_MOESM2_ESM.pdf]

Team Emergency Assessment Measure (TEAM)

Introduction

This non- technical skills questionnaire has been designed as an observational rating score for valid, reliable and feasible ratings of emergency medical teams (e.g. resuscitation and trauma teams). The questionnaire should be completed by expert clinicians to enable accurate performance rating and feedback of leadership, team work, situation awareness and task management. Rating prompts are included where applicable. The following scale should be used for each rating:

Never/Hardly ever	seldom	About as often as not	Often	Always/Nearly always
0	1	2	3	4

Team Identification

Date: Time: Place:

Team Leader: Team:

**Leadership: it is assumed that the leader is either designated, has emerged or is the most senior - if no leader emerges allocate a '0' to question 1 and 2.**	**0**	**1**	**2**	**3**	**4**
**1.The team leader let the team know what was expected of them through direction and command**	☐	☐	☐	☐	☐
**2. The team leader maintained a global perspective**<br>*Prompts: Monitoring clinical procedures and the environment? Remaining 'hands off' as applicable? Appropriate delegation.*	☐	☐	☐	☐	☐
**Team Work: ratings should include the team as a whole i.e. the leader and the team as a collective (to a greater or lesser extent).**	**0**	**1**	**2**	**3**	**4**
**3. The team communicated effectively**<br>*Prompts: Verbal, non-verbal and written forms of communication?*	☐	☐	☐	☐	☐
**4. The team worked together to complete the tasks in a timely manner**	☐	☐	☐	☐	☐
**5. The team acted with composure and control**<br>*Prompts: Applicable emotions? Conflict management issues?*	☐	☐	☐	☐	☐
**6. The team morale was positive**<br>*Prompts: Appropriate support, confidence, spirit, optimism, determination?*	☐	☐	☐	☐	☐
**7. The team adapted to changing situations**<br>*Prompts: Adaptation within the roles of their profession?*<br>*Situation changes: Patient deterioration? Team changes?*	☐	☐	☐	☐	☐
**8. The team monitored and reassessed the situation**	☐	☐	☐	☐	☐
**9. The team anticipated potential actions**<br>*Prompts: Preparation of defibrillator, drugs, airway equipment?*	☐	☐	☐	☐	☐
**Task Management:**	**0**	**1**	**2**	**3**	**4**
**10. The team prioritised tasks**	☐	☐	☐	☐	☐
**11.The team followed approved standards and guidelines**<br>*Prompt: Some deviation may be appropriate?*	☐	☐	☐	☐	☐

**Overall:**	**1**	**2**	**3**	**4**	**5**	**6**	**7**	**8**	**9**	**10**
**12. On a scale of 1-10 give your global rating of the team's non-technical performance**	☐	☐	☐	☐	☐	☐	☐	☐	☐	☐

Comments:____

____
