# Supplementary material for: Video-based feedback as a method for training rural healthcare workers to manage medical emergencies: a pilot study
Source: BMC Med Educ. 2017 Aug 31;17:149. doi: 10.1186/s12909-017-0975-3 (PMC5580284; doi:10.1186/s12909-017-0975-3)
Supplement: Supplementary file 3 — Focus Group Discussion (DOCX 13 kb) [file 12909_2017_975_MOESM3_ESM.docx]

**Focus group discussion- Team Feedback/confidence assessment**

1. Do you feel that you know more now than you did before the training? Please explain
2. Do you feel more confident about managing an emergency scenario? Why do you feel this way?
3. What aspects of the course contributed most to your learning, and how?
4. Do you feel that you will be able to use what you learnt today in other emergency scenarios? Why/why not?
5. Is there anything else you would like to share about your training experience today?
